# Atrial Fibrillation, Stroke, and Silent Cerebrovascular Disease: A Population-based MRI Study

**DOI:** 10.1212/WNL.0000000000012675

**Published:** 2021-10-19

**Authors:** Lina Rydén, Simona Sacuiu, Hanna Wetterberg, Jenna Najar, Xinxin Guo, Silke Kern, Anna Zettergren, Sara Shams, Joana B. Pereira, Lars-Olof Wahlund, Eric Westman, Ingmar Skoog

**Affiliations:** From the Institute of Neuroscience and Physiology (L.R., S.Sacuiu, H.W., J.N., X.G., S.K., A.Z., I.S.), Sahlgrenska Academy, Centre for Ageing and Health at the University of Gothenburg; Department of Psychiatry Cognition and Old Age Psychiatry (L.R., S.S., J.N., S.K., I.S.), Sahlgrenska University Hospital, Region Västra Götaland, Mölndal; Department of Mood Disorders (X.G.), Sahlgrenska University Hospital, Region Västra Götaland, Göteborg; Division of Clinical Geriatrics (S.Shams, J.B.P., L.-O.W., E.W.), Centre for Alzheimer Research, Department of Neurobiology, Care Sciences, and Society, Karolinska Institutet, Stockholm, Sweden; Department of Radiology (S.S.), Stanford, CA; and Clinical Memory Research Unit (J.B.P.), Department of Clinical Sciences, Malmö, Lund University, Sweden.

## Abstract

**Background and Objectives:**

Atrial fibrillation (AF) has been associated with cognitive decline and dementia. However, the mechanisms behind these associations are not clear. Examination of cerebrovascular pathology on MRI may shed light on how AF affects the brain. This study aimed to determine whether AF is associated with a broad range of cerebrovascular diseases beyond the well-known association with symptomatic stroke, including silent infarcts and markers of small vessel disease, i.e., cerebral microbleeds (CMBs), white matter hyperintensities (WMHs), and lacunes, in a population-based sample of 70-year-olds.

**Methods:**

Data were obtained from the Gothenburg H70 Birth Cohort Studies, in which individuals are invited based on birthdate. This study has a cross-sectional design and includes individuals born in 1944 who underwent structural brain MRI in 2014 to 2017. AF diagnoses were based on self-report, ECG, and register data. Symptomatic stroke was based on self-report, proxy interviews, and register data. Brain infarcts and CMBs were assessed by a radiologist. WMH volumes were measured on fluid-attenuated inversion recovery images with the Lesion Segmentation Tool. Multivariable logistic regression was used to study the association between AF and infarcts/CMBs, and multivariable linear regression was used to study the association between AF and WMHs.

**Results:**

A total of 776 individuals were included, and 65 (8.4%) had AF. AF was associated with symptomatic stroke (odds ratio [OR] 4.5, 95% confidence interval [CI] 2.1–9.5) and MRI findings of large infarcts (OR 5.0, 95% CI 1.5–15.9), lacunes (OR 2.7, 95% CI 1.2–5.6), and silent brain infarcts (OR 3.5; 95% CI 1.6–7.4). Among those with symptomatic stroke, individuals with AF had larger WMH volumes (0.0137 mL/total intracranial volume [TIV], 95% CI 0.0074–0.0252) compared to those without AF (0.0043 mL/TIV, 95% CI 0.0029–0.0064). There was no association between AF and WMH volumes among those without symptomatic stroke. In addition, AF was associated to CMBs in the frontal lobe.

**Discussion:**

AF was associated with a broad range of cerebrovascular pathologies. Further research is needed to establish whether cerebrovascular MRI markers can be added to current treatment guidelines to further personalize anticoagulant treatment in patients with AF and to further characterize the pathogenetic processes underlying the associations between AF and cerebrovascular diseases, as well as dementia.

Atrial fibrillation (AF) is the most common clinically relevant cardiac arrhythmia, affecting 1% to 4% of the adult population and >13% of persons ≥80 years of age.^1^ AF has been associated with stroke,^2^ dementia,^3^ and mortality, but the mechanisms behind these associations, in the absence of cardiac embolism, are not clear. Suggested mechanisms include systemic inflammation and brain hypoperfusion due to reduced cardiac output.^4^ In addition, AF shares several risk factors with both stroke and dementia.^5^

Cerebral small vessel disease (cSVD) affects small arteries, arterioles, capillaries, and small veins^6^and has been related to cognitive decline, vascular dementia, and stroke.^6,7^ Different pathologic processes may lead to cSVD such as arteriosclerosis, cerebral amyloid angiopathy (CAA), venous collagenosis, and inflammation.^6^ Markers of cSVD on brain MRI includes white matter hyperintensities (WMHs), lacunes of presumed vascular origin, and cerebral microbleeds (CMBs).^7^ The most established risk factors for WMHs are hypertension and advanced age,^8^ but other cardiovascular risk factors may also play a role.^9^ Lacunes are highly correlated with WMHs^6^ and are associated with advanced age and vascular risk factors such as hypertension, smoking, and diabetes.^10^ For CMBs, different topography has been linked to different etiologies; lobar CMBs have been associated with CAA, while CMBs in the infratentorial and deep brain regions have been associated with atherosclerosis and hypertension.^11^ Because AF is associated with both symptomatic stroke and silent infarcts (i.e., infarcts on brain imaging without clinical symptoms), it is important to also consider the influence of stroke and silent infarcts when studying the effects of AF on the brain.

This study aimed to determine whether AF is associated with a broad range of cerebrovascular diseases, beyond the well-known association with symptomatic stroke, including silent infarcts and markers of small vessel disease, i.e., CMBs, WMHs, and lacunes, in a population-based sample of 70-year-olds. In addition, we aimed to determine whether the association with WMHs was affected by the presence of stroke and anticoagulant treatment and age at AF onset.

## Methods

### Participants

Data were obtained from the population-based Gothenburg H70 Birth Cohort Studies (the H70 studies).^12^ The present study has a cross-sectional design, and in total, 1,667 men and women living in Gothenburg, Sweden, born in 1944 on birthdates ending with 0, 2, 5, and 8 were invited to participate. A total of 1,203 individuals (559 men, 644 women; response rate 72%) were examined in 2014 to 2016,^12^ and 791 individuals (414 women, 377 men) underwent structural brain MRI in 2014 to 2016. After the exclusion of individuals with multiple sclerosis, normal-pressure hydrocephalus, Parkinson disease, and valvular diseases (mitral valve disease or artificial heart valves), a total of 776 individuals remained for analyses.

### Standard Protocol Approvals, Registrations, and Patient Consents

The study was approved by the Regional Ethical Review Board in Gothenburg. Informed consent was obtained from all participants or their relatives if the participant was unable to provide informed consent.

### General Examinations

Examinations were conducted by research nurses or medical doctors. Participants were asked about present and past diseases and medications. The interviews also included questions about sudden onset of focal neurologic symptoms, duration of symptoms, and admission to hospital due to stroke. Physical examinations included anthropometry, blood pressure, blood sampling, and ECG. The ECG was coded according to the Minnesota Code (MC), and blood pressure was measured in the right arm after 5 minutes of rest in the seated position with a manual sphygmomanometer. DNA was extracted from blood samples according to standard procedures.^12^

Neuropsychiatric examinations were performed by psychiatric research nurses or medical doctors and included questions about psychiatric disorders and symptoms and assessments of signs of dementia, including cognitive tests. Proxy interviews were performed by a psychologist or research nurse and included questions about history of stroke symptoms and signs and symptoms of dementia.

Additional data were obtained from the National Patient Register (NPR), containing hospital discharge diagnoses and specialized outpatient care coded according to ICD10-SE.

### Brain Imaging

All participants were scanned on a 3.0T Philips Achieva system (Philips Medical Systems, Best, the Netherland) using a protocol including T1, T2, fluid-attenuated inversion recovery, T2*, and diffusion-weighted imaging.^12^ Large infarcts (>15 mm), lacunes (3–15 mm), intracerebral hemorrhages, and CMBs were assessed by an experienced radiologist (S.Shams)^13^ blinded to clinical data. Lacunes and CMBs were defined according to the Standards for Reporting Vascular Changes on Neuroimaging.^7^ CMBs were recorded by location, that is, lobar (temporal, parietal, frontal, occipital) or deep/infratentorial. The fluid-attenuated inversion recovery sequence, which was used in WMH measurements, was acquired with the following parameters: 2.0-mm isotropic resolution sagittal slices, echo time 280 milliseconds, repetition time 4,800 milliseconds, field of view 250 × 250 mm^2^, flip angle 90°, and 140 slices.

WMH volumes were automatically segmented with the Lesion Segmentation Tool 2.0.15 using a lesion prediction algorithm and running under the statistical parametric mapping software.^14,15^ A quality control of the lesion segmentation output was applied, and those with invalid segmentation (n = 13) were excluded, leaving 763 individuals for WMH volume analyses. Data were managed and processed through TheHiveDB system.^16^

### Definition of AF and Stroke

The diagnosis of AF was based on self-report, ECG (MC 8-3), and the NPR (ICD10-SE: I48) and included all individuals with a history of AF. Age at AF onset was dichotomized to <65 vs ≥65 years of age and was based on the earliest reported date of AF from self-report, ECG, or the NPR. The diagnosis of symptomatic stroke was based on self- or proxy-reported history of stroke symptoms and the NPR (ICD10-SE I60-I61, I62.9, I63.0–I63.5, I63.8, I63.9, I64, I69.0–I69.1, I69.3–I69.4), as described previously.^17^ The criteria for stroke according to self- and proxy reports required a clear history of acute focal neurologic symptoms (including aphasia) lasting for >24 hours.

Large infarcts and lacunes on MRI could be either symptomatic or silent. Silent brain infarcts were defined as large infarcts or lacunes on MRI in an individual without history of symptomatic stroke.

### Definition of Covariates

Education level was defined as mandatory (corresponding to 7–8 years) or less vs more than mandatory. Heart diseases included heart failure and myocardial infarction. Heart failure was defined through self-report or the NPR (ICD10-SE I11.0, I13.0, I13.2, I50). Myocardial infarction was defined through self-report, the NPR (ICD10-SE I21–I23, I24.1, I25.2, I25.6), or major or moderate Q waves on ECG (MC 1.1–1.2, excluding 1.2.6 and 1.2.8). Hypertension was defined as present medical treatment for hypertension, systolic blood pressure ≥150 mm Hg, or diastolic blood pressure ≥90 mm Hg as suggested by the Eight Joint National Committee in 2014.^18^ Diabetes mellitus was defined as present treatment with insulin or antidiabetic drug, fasting glucose ≥7.0 mmol/L, or nonfasting glucose ≥11.1 mmol/L. Hypercholesterolemia was defined as present treatment with lipid-lowering medication, total cholesterol >6.2 mmol/L, low-density lipoprotein cholesterol ≥4.1 mmol/L, triglycerides >5.6 mmol/L, or high-density lipoprotein cholesterol <1.0 mmol/L in men or <1.3 mmol/L in women.^19^ Smoking was based on self-report and defined as never smoker, past smoker, or current smoker. Body mass index (BMI) was defined as weight in kilograms divided by the square of height in meters. Alcohol risk consumption was defined according to the National Institute on Alcohol Abuse and Alcoholismguidelines^20^ as >98 g alcohol/wk based on self-reported consumption during the last month.

### Definition of Diagnoses Used for Exclusion

Dementia was diagnosed according to the DSM-III-R using information from psychiatric examinations and proxy reports, as described previously.^21^ Diagnoses of multiple sclerosis, normal-pressure hydrocephalus, and Parkinson disease were based on self-report and the NPR (ICD10-SE G20, G35, and G91). Valvular diseases (mitral valve disease or artificial heart valves) were obtained from the NPR (ICD10-SE I34, Z952-954, I05, I08, Q23). TIA was based on self- or proxy-reported history of stroke symptoms lasting <24 hours and the NPR (ICD10-SE G45.0–G45.3, G45.8–G45.9).

### Statistical Analyses

Independent-sample *t* test was used for continuous variables, and Fisher exact test and Pearson χ^2^ test were used for categorical variables when comparing participant characteristics by brain MRI and AF status.

Binary logistic regression was used to study the association between AF and stroke (i.e., symptomatic strokes, large infarcts, lacunes, and silent brain infarcts). To study whether the relation between AF and stroke was affected by sex, interactions for AF and sex were performed. Stratified analyses based on sex were performed regardless of the significance level of the interaction term. Binary logistic regression was used to study the association between AF and CMBs, by location and run separately for men but not for women because only 1 woman with AF had CMBs.

Linear regression was used to study the association between AF and WMH volumes. WMH volumes were adjusted for total intracranial volume (TIV) by dividing WMH volume by TIV. A logarithmic transformation was applied to the adjusted WMH volumes due to nonnormal distribution of the residuals. To study whether the relation between AF and WMHs was affected by sex or stroke status, interactions for AF and sex and for AF and stroke were performed. Stratified analyses based on sex were performed regardless of the significance level of the interaction term. In addition, the sample was stratified into 4 groups based on AF and stroke status; (1) no AF and no stroke, (2) AF and no stroke, (3) no AF and stroke, and (4) AF and stroke. These analyses were corrected for multiple comparison with Bonferroni correction.

Analyses including stroke/infarcts and WMHs were repeated after the exclusion of individuals with dementia. For analyses including silent brain infarcts, individuals with a history of symptomatic stroke (n = 44) were excluded. The analyses including silent brain infarcts were repeated after the additional exclusion of individuals with TIA. Sex, education, heart disease, hypertension, diabetes, hypercholesterolemia, BMI, smoking, alcohol risk consumption, and the *APOE* ε4 allele were considered possible covariates. In analyses including WMHs and CMBs, stroke and infarcts were also considered possible covariates. Purposeful variable selection was applied in all adjusted models^22^; that is, all potential covariates were included in univariable models and thereafter in the multivariable models if the values of *p* were <0.2. In multivariable analyses, covariates with the largest *p* values were removed one by one until all remaining covariates had *p* < 0.2. The estimate of the predictors was controlled after each removal, and the covariate was reentered if the estimate changed >15%. Individuals with missing data were omitted from the analyses.

### Data Availability

The data that support the findings of this study are available from the corresponding author on reasonable request.

## Results

Participants in brain MRI (n = 791) more often had more than mandatory education (88% vs 80%), less often had dementia (1.6% vs 4.2%), and less often were current smokers (8% vs 13%) compared to nonparticipants (n = 412) (Table 1). Among participants in the present study (n = 776 after exclusion of individuals with multiple sclerosis, normal-pressure hydrocephalus, Parkinson disease, and valvular diseases), individuals with AF (n = 65) were more often men and more often had heart disease, diabetes, hypercholesterolemia, higher CHA2DS2-VASc score, and anticoagulant treatment than those without AF (n = 711) (Table 2).

**Table 1:**
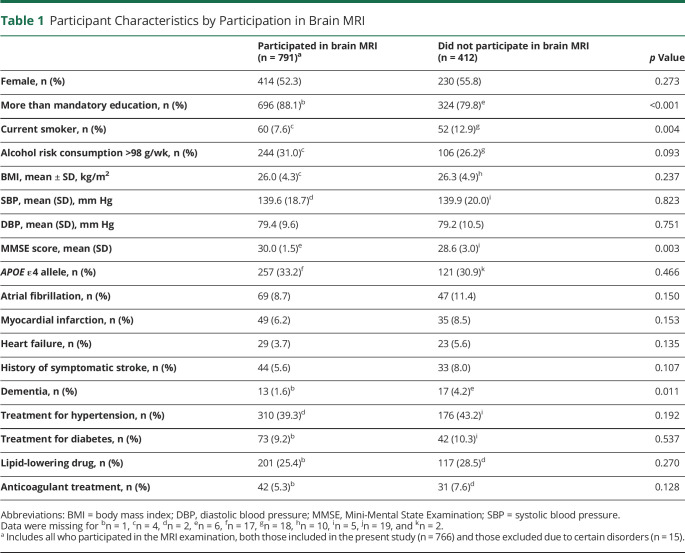
Participant Characteristics by Participation in Brain MRI

**Table 2:**
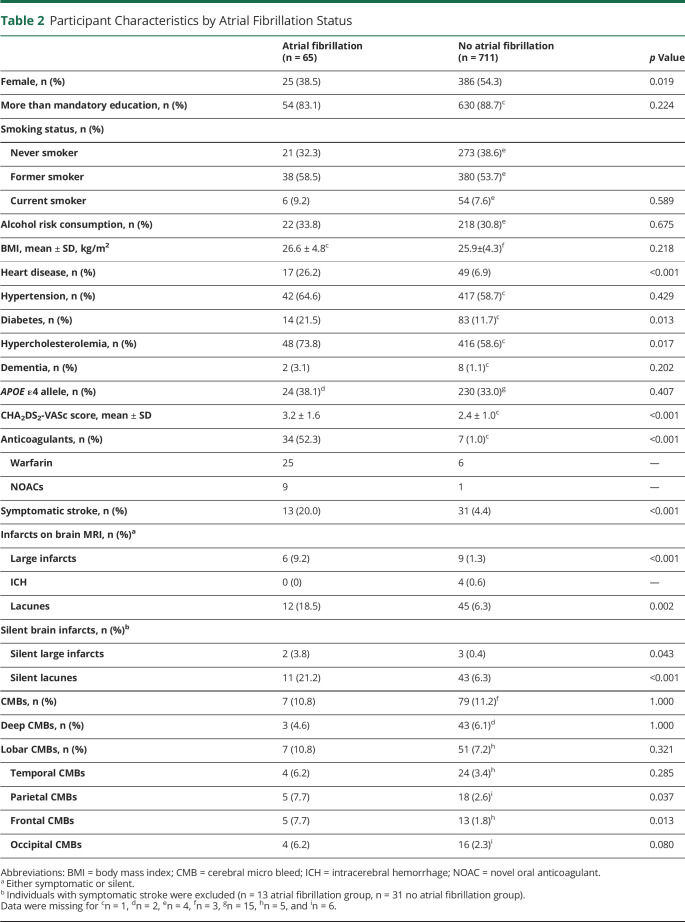
Participant Characteristics by Atrial Fibrillation Status

### AF and Stroke

AF was associated with a history of symptomatic stroke (odds ratio [OR] 4.5, 95% confidence interval [CI] 2.1–9.5), large infarcts (OR 5.0, 95% CI 1.5–5.9), lacunes (OR 2.7, 95% CI 1.2–5.6), and silent brain infarcts (OR 3.5, 95% CI 1.6–7.4) (Table 3). Excluding individuals with dementia (and TIA for the analysis of silent brain infarcts) did not affect the results. No interactions were found between AF and sex in relation to symptomatic stroke (*p* = 0.399 in the adjusted model), large infarcts (*p* = 0.932), lacunes (*p* = 0.313), or silent brain infarcts (*p* = 0.410). However, after exclusion of individuals with dementia, the interaction for AF and sex in relation to silent brain infarcts had a value of *p* = 0.121. In stratified analyses by sex, AF was associated with symptomatic stroke, large infarcts, lacunes, and silent brain infarcts in men. In women, there were no significant associations between AF and symptomatic stroke, lacunes, and silent brain infarcts, but there was a trend toward an association between AF and large infarcts.

**Table 3:**
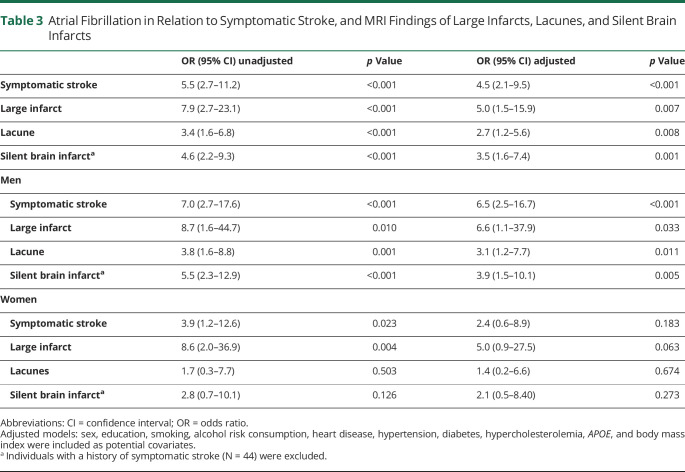
Atrial Fibrillation in Relation to Symptomatic Stroke, and MRI Findings of Large Infarcts, Lacunes, and Silent Brain Infarcts

### AF and WMH Volumes

Individuals with AF had larger WMH volumes than those without after adjustment for TIV. However, the association was weakened after additional adjustment for cardiovascular risk factors and prevalent heart diseases. After additional adjustment for symptomatic stroke or MRI findings of infarcts, the association disappeared (Table 4). No interaction was found for AF and sex in relation to WMH volumes (*p* = 0.654 in the adjusted model including symptomatic stroke). An interaction was found for AF and symptomatic stroke in relation to WMH volumes (*p* = 0.003 in the adjusted model) but not for AF and lacunes (*p* = 0.199) or AF and silent brain infarcts (*p* = 0.912). Individuals with AF and present anticoagulant treatment had larger WMH volumes than individuals without AF after adjustment for cardiovascular risk factors, prevalent heart diseases, and symptomatic stroke. Individuals with AF but without anticoagulation did not have larger WMH volumes compared to individuals without AF (Table 5). Among those with AF and anticoagulant treatment, 85% (n = 29 of 34) had a CHA_2_DS_2_-VASc score >1 (for men) or >2 (for women). Among those with AF but without anticoagulant treatment, 73% (n = 22 of 30) had a CHA_2_DS_2_-VASc >1 (for men) or >2 (for women). Age at AF onset did not influence WMH volumes after adjustments (Table 5). Excluding individuals with dementia did not affect the results.

**Table 4:**
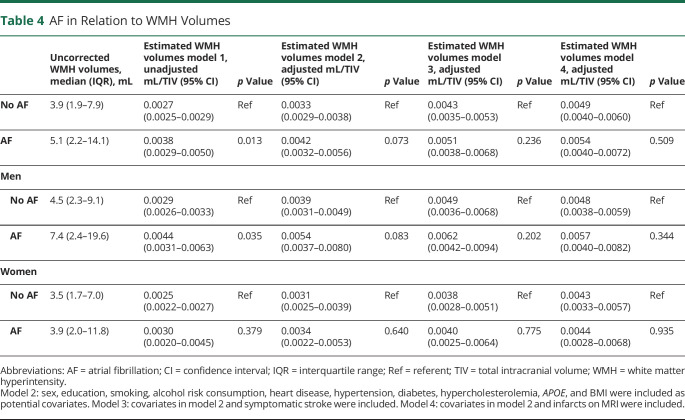
AF in Relation to WMH Volumes

**Table 5:**
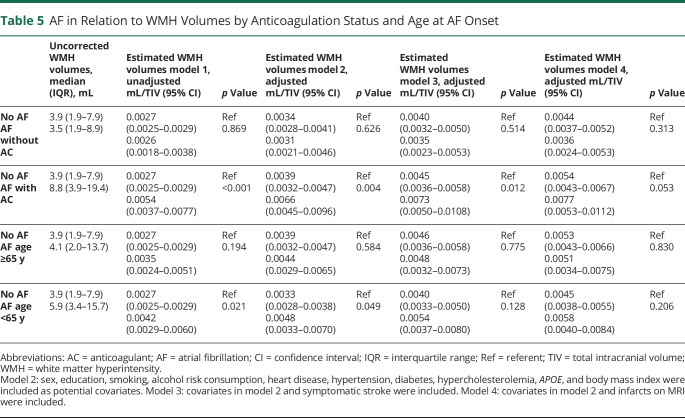
AF in Relation to WMH Volumes by Anticoagulation Status and Age at AF Onset

### AF, History of Symptomatic Stroke, and WMH Volumes

Figure, A shows the estimated WMH volumes divided by TIV in 4 groups: (1) no AF and no symptomatic stroke, (2) AF and no symptomatic stroke, (3) no AF and symptomatic stroke, and (4) AF and symptomatic stroke. Group 4 (AF and symptomatic stroke) had larger WMH volumes compared to all other groups. There were no other differences between the groups. Excluding individuals with dementia did not affect the results.

**Figure:**
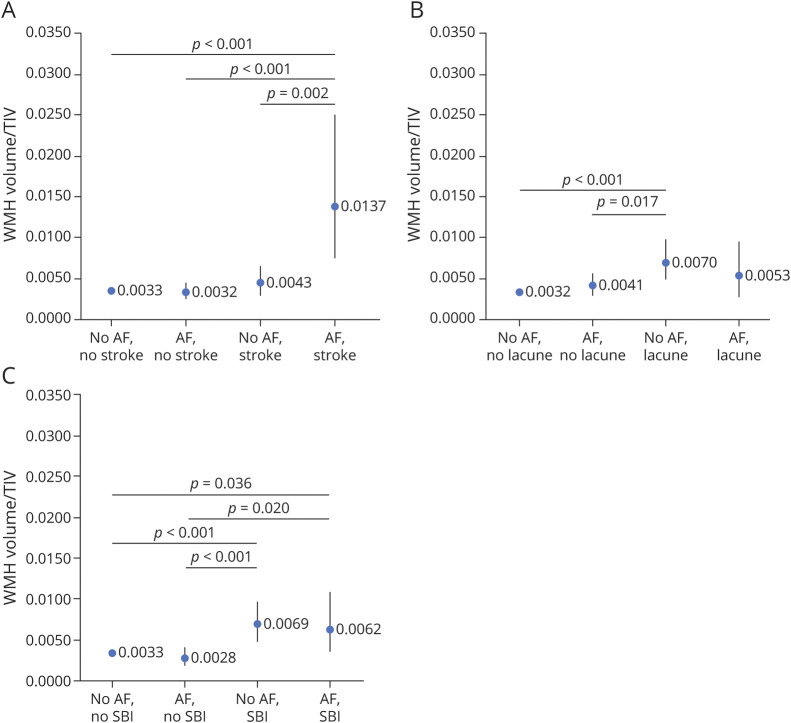
Estimated WMH Volume as a Fraction of TIV By AF and Stroke Status Estimated white matter hyperintensity (WMH) volume as a fraction of total intracranial volume (TIV) divided into 4 groups by (A) atrial fibrillation (AF) and history of symptomatic stroke status, (B) AF and silent brain infarct (SBI) status, and (C) AF and lacune status. Sex, education, smoking, alcohol risk consumption, heart disease, hypertension, diabetes, hypercholesterolemia, *APOE*, and body mass index were included as potential covariates. Values of *p* <0.05 are shown; *p* < 0.008 is considered statistically significant after Bonferroni correction.

**Table 6:**
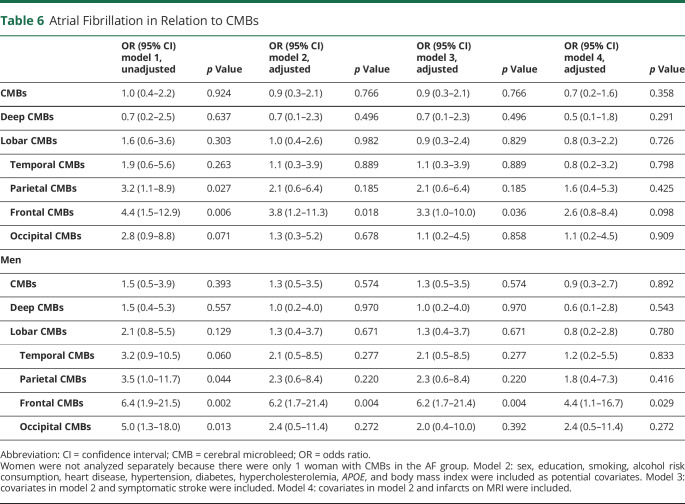
Atrial Fibrillation in Relation to CMBs

### AF, Lacunes, and WMH Volumes

Figure, B shows the estimated WMH volumes in 4 groups: (1) no AF and no lacune, (2) AF and no lacune, (3) no AF and lacune, and (4) AF and lacune. Group 3 (no AF and lacune) had larger WMH volume compared to group 1 (no AF and no lacune). Group 4 did not differ statistically from the other groups. Excluding individuals with dementia did not affect the results.

### AF, Silent Brain Infarcts, and WMH Volumes

Figure, C shows the estimated WMH volumes in 4 groups: (1) no AF and no silent brain infarct, (2) AF and no silent brain infarct, (3) no AF and silent brain infarct, and (4) AF and silent brain infarct. Group 3 (no AF and silent brain infarct) had larger WMH volume compared to both groups 1 (no AF and no silent brain infarct) and 2 (AF and no silent brain infarct). Group 4 (AF and silent brain infarct) did not differ statistically from the other groups, but the estimated WMH volume was similar to that of group 3. There were no other differences between the groups. Excluding individuals with dementia or TIA did not affect the results. There were too few individuals with large infarcts on brain MRI for subgroup analyses.

### AF and Cerebral Microbleeds

AF was not associated with the presence of CMBs, lobar CMBs, or deep/infratentorial CMBs (Table 6). However, after the CMBs were divided by lobe, AF was associated with CMBs in the parietal, frontal, and occipital lobes in the unadjusted models. In the adjusted models, including symptomatic stroke, AF was associated with CMBs only in the frontal lobe (OR 3.3, 95% CI 1.0–10.0). After adjustment for infarcts on MRI, the association became weaker (OR 2.6, 95% CI 0.8–8.4). Among men, AF was associated with CMBs in the frontal lobe in the adjusted model including symptomatic stroke (OR 6.2, 95% CI 1.7–21.4) and infarcts on MRI (OR 4.4, 95% CI 1.1–16.7). Because only 1 woman with AF had CMBs, we did not analyze the association between AF and CMBs among women.

## Discussion

We found that AF was associated with history of symptomatic stroke, large infarcts, lacunes, and silent brain infarcts. AF was associated with larger WMH volumes in individuals with symptomatic stroke but not in those without. However, despite the fact that silent brain infarcts and lacunes were associated with larger WMH volumes, AF did not affect these relations. In addition, individuals with AF and anticoagulant treatment had larger WMH volumes than individuals without AF, while individuals with AF but without anticoagulant treatment did not have larger WMH volumes than those without AF. AF was not associated with lobar or deep/infratentorial CMBs except in the frontal lobe, where microbleeds were more common in individuals with AF.

We found that AF was related to both history of symptomatic stroke and large infarcts on brain MRI after adjusting for demographic factors, cardiovascular risk factors, and comorbid conditions. No interactions were found for AF and sex in relation to symptomatic stroke or large infarcts on MRI. Traditionally, the dysrhythmia in AF has been regarded as the major cause of embolic stroke, with an almost 5-fold increased risk of overall stroke.^2^ In contrast, AF did not increase overall stroke risk in individuals without cardiovascular comorbid conditions in a recent large population-based study.^23^ Instead, cardiovascular comorbid conditions increased risk of stroke independently of AF.^23^ It has been suggested that aging and vascular risk factors cause abnormal atrial substrate that may result in both AF and stroke. Once AF develops, it might further increase stroke risk.^24^ Whether AF on its own has a causal relationship with stroke is still being discussed.^23-25^ In addition, AF is often asymptomatic and therefore not diagnosed until complications, for example, stroke and heart failure,^26^ occur, which may overestimate the association between AF and stroke.

After excluding individuals with history of symptomatic stroke, we found that AF was related to a >3-fold increase in the odds of silent brain infarcts after adjustment for several confounding variables. This is in line with a meta-analysis from 2014 showing that AF was related to a >2-fold increase in the odds of silent brain infarcts.^27^ Because most silent brain infarcts are lacunes,^28^ it was not surprising that we also found an association between AF and lacunes on brain MRI. The most established risk factors for silent brain infarcts are hypertension and advanced age, but silent brain infarcts have also been associated with different cardiac diseases (e.g., AF, cardiomyopathy, and patent foramen ovale) and procedures such as coronary artery bypass graft surgery, indicating that cardiac factors may play a role.^28^ No interactions were found for AF and sex in relation to lacunes or silent brain infarcts. However, after stratification for sex, significant associations between AF and lacunes and silent brain infarcts were observed only in men. We have previously reported that the association between AF and dementia in a sample without symptomatic stroke was seen only in men in both the unadjusted and adjusted models.^3^ In contrast, female sex is a well-recognized risk factor for stroke in patients with AF and is included as such in guidelines of stroke risk (i.e., CHA_2_DS_2_-VASc score). Because we did not find an interaction for AF and sex in relation to stroke, we cannot make any conclusions regarding sex differences in the relations.

Our finding that the association between AF and WMHs disappeared after taking stroke and cardiovascular risk factors into account is in line with results from the Mayo Clinic Study of Aging^29^ and the Atherosclerosis Risk in Communities Neurocognitive Study.^30,31^ In addition, our finding that AF was not associated with WMHs in individuals without a history of stroke is in line with the Framingham offspring study^32^ and the German AF Competence NETwork Study.^33^ However, in our study, individuals with both AF and anticoagulant treatment had larger WMH volumes than individuals without AF after adjustments, including cardiovascular risk factors, heart disease, and symptomatic stroke, while individuals with AF but no anticoagulant treatment did not have larger WMH volumes than individuals without AF. However, this could be due to confounding by indication because current treatment guidelines for AF (CHA_2_DS_2_-VASc score) advocate anticoagulant treatment for individuals with more comorbid conditions such as congestive heart failure, hypertension, diabetes, stroke, and vascular diseases. Therefore, even if multiple adjustments were performed, including risk factors in the CHA_2_DS_2_-VASc score, individuals with both AF and anticoagulant treatment might have more severe cardiovascular disorders and more comorbid conditions than those with AF alone. We cannot, however, exclude the possibility that anticoagulant treatment may lead to more WMHs due to, for example, minor subclinical bleedings. We take note of a recent study reporting that in individuals with intracerebral hemorrhage, those taking anticoagulants did not have higher WMH burden than those not taking anticoagulants after adjustments for potential confounders.^34^ It is worth noticing that there were few individuals on novel oral anticoagulant compared to warfarin in individuals with AF in our study.

We found an interaction between AF and symptomatic stroke in relation to WMH volumes. Thus, AF was related to larger WMH volumes in persons with a history of symptomatic stroke but not in those without. In contrast to our results, studies on patients with acute ischemic stroke have not found a higher WMH burden in individuals with AF compared to those without AF.^35-38^ This discrepancy suggests that AF in itself does not lead to WMHs but accentuates the development of WMHs initiated by the stroke. One explanation might be that a brain affected by stroke is more vulnerable to, for example, hypoperfusion due to AF. AF did not affect the associations between lacunes or silent brain infarcts and WMH volumes. One explanation may be that silent brain infarcts and lacunes often affect small brain areas, thus making the brain less vulnerable to hypoperfusion due to AF. However, due to the small number of individuals with both AF and stroke, our results should be interpreted cautiously.

In comparisons of WMH volumes using automated imaging processing tools (i.e., the Lesion Segmentation Tool) with the Fazekas scale, a cutoff of 0.00496 mL WMH/TIV has been proposed to classify low and high Fazekas WMH burden, indicating a clinically relevant burden.^14^ In our study, only those with both AF and a history of symptomatic stroke had estimated WMH volumes above this cutoff, while the other groups had estimates around or below this value. Because comorbid conditions may exacerbate clinical outcomes, it is useful to study risk factors and diseases in combinations. For example, it has been suggested that aging and hypertension exacerbate WMH burden and inhibit WMH repair in patients with stroke and that this needs to be accounted for in clinical trials to improve stroke outcomes.^39^ This study showed that AF also is a risk marker of WMHs in individuals with a history of symptomatic stroke. Because WMHs are associated with unfavorable outcomes such as dementia, depression, and stroke,^40^ individuals with both AF and a history of symptomatic stroke might be particularly vulnerable to unfavorable outcomes.

We did not find any association between AF and lobar or deep/infratentorial microbleeds in multivariable analyses except in the frontal lobe, where microbleeds were more common in individuals with AF than in those without. In contrast, a case-control study from Japan^41^ found a higher prevalence of CMBs in patients with AF compared to controls without AF. However, cases differed from controls regarding sex, hypertension, diabetes, and medications. Thus, factors other than AF might have contributed to the higher CMB prevalence. CMB topography has been linked to different pathologies; lobar CMBs are associated with CAA, while deep and infratentorial CMBs are associated with atherosclerosis and hypertension.^11^ In addition, it has been shown that individuals with Alzheimer dementia have a predominant lobar topography.^13^ Our finding of an association between AF and CMBs in the frontal lobe could therefore be due to CAA. However, it has been suggested that CAA-related CMBs have a posterior predominance.^11,42^ The findings that frontal CMBs were more common in individuals with AF and that we did not find an association between AF and other lobar or deep/infratentorial CMBs need to be interpreted cautiously because there were few cases with both AF and CMBs.

Our study has several strengths such as the large population-based sample with MRI, comprehensive examinations performed by health professionals, and the use of multiple sources, including self- and proxy reports, register, physical examinations, and biomarkers. We have previously validated self-reported AF diagnoses in the H70 studies against register data and found a substantial agreement between the 2 sources (κ = 0.61).^43^ However, some limitations should also be recognized. First, this study has a cross-sectional design, which does not allow us to assess causal relationships. Second, AF may remain undiagnosed in asymptomatic individuals. Our examinations included ECGs to detect silent AF; however, for example, 24-hour Holter monitoring can increase AF detection.^44^ Third, sample size limits the possibility of doing all warranted subgroup analyses. Fourth, we were not able to examine regional patterns of WMHs. Other studies have reported associations between AF and WMHs in the deep and subcortical white matter^45^ or periventricular white matter^46^ only. Fifth, our study only includes 70-year-olds, limiting generalizability to other age groups.

We found that AF was associated with a history of symptomatic stroke and several brain pathologic markers such as large infarcts, lacunes, and silent brain infarcts. AF was associated with larger WMH volumes in individuals with symptomatic stroke but not in those without. In addition, AF was not associated with lobar or deep/infratentorial CMBs except in the frontal lobe, where microbleeds were more common in individuals with AF.

Further research is needed to establish whether cerebrovascular MRI markers can be used as a complement to current treatment guidelines to further personalize anticoagulant treatment in patients with AF and to further characterize the pathogenetic processes underlying the associations between AF and cerebrovascular diseases, as well as dementia.

## Glossary

AF: atrial fibrillation
BMI: body mass index
CAA: cerebral amyloid angiopathy
CI: confidence interval
CMB: cerebral microbleed
cSVD: cerebral small vessel disease
DSM-III-R: *Diagnostic and Statistical Manual of Mental Disorders,* 3rd edition, revised
ICD: *International Classification of Diseases*
MC: Minnesota Code
NPR: National Patient Register
OR: odds ratio
TIV: total intracranial volume
WMH: white matter hyperintensity
